# Network Analysis of Convergent and Specific Molecular Pathways of Nutraceuticals with Antioxidant and Neuroprotective Potential in Glaucoma

**DOI:** 10.3390/antiox15040445

**Published:** 2026-04-02

**Authors:** Pavlina Teneva, Sylvia Stamova, Kaloyan Varlyakov, Neli Ermenlieva, Emilia Georgieva, Todorka Kostadinova

**Affiliations:** 1 Department of Health Care, Medical College, Trakia University, 6000 Stara Zagora, Bulgaria; kaloyan.varlyakov@trakia-uni.bg; 2 Department of Pharmaceutical Chemistry, Pharmacy Faculty, Medical University of Varna, 9000 Varna, Bulgaria; 3 Department of Microbiology and Virology, Faculty of Medicine, Medical University of Varna, 9000 Varna, Bulgaria; neli.ermenlieva@mu-varna.bg; 4 Training Sector “Medical Laboratory Technician”, Medical College of Varna, Medical University of Varna, 9000 Varna, Bulgaria; emiliya.georgieva@mu-varna.bg; 5 Department of Economics and Health Care Management, Faculty of Public Health, Medical University Prof. Dr. P. Stoyanov, 9002 Varna, Bulgaria; kostadinova@mu-varna.bg

**Keywords:** glaucoma, antioxidant nutraceuticals, network pharmacology, protein–protein interaction (PPI), neuroprotection, oxidative stress

## Abstract

Optic neuropathy represents a leading cause of irreversible vision loss, in which oxidative stress, chronic inflammation, dysregulated lipid metabolism, and mitochondrial dysfunction contribute to the progressive degeneration of retinal ganglion cells (RGCs). In recent years, a number of nutraceuticals have been investigated as potential neuroprotective agents; however, the molecular mechanisms through which they exert their effects remain incompletely understood and are often considered in isolation. In the present in silico study, an integrative network-based approach was applied for a systematic analysis of the predicted molecular targets of selected nutraceuticals with antioxidant and anti-inflammatory potential. By combining target prediction, protein–protein interaction analysis, and functional enrichment, their functional convergence was assessed in the context of optic nerve pathophysiology. The results indicate that, despite their chemical and functional heterogeneity, the investigated nutraceuticals do not act through fully independent mechanisms but instead converge on interconnected regulatory axes. In particular, lipid–inflammatory signaling, epigenetic and stress-adaptive mechanisms, as well as nuclear-receptor mediated transcriptional regulation emerged as key pathways. These pathways form integrated molecular models potentially determining cellular susceptibility to injury and the adaptive capacity of RGCs. In conclusion, the present analysis provides a systems-level framework for understanding the neuroprotective potential of nutraceuticals, highlighting the importance of network convergence and multi-target activity. The obtained results support the conceptual shift from isolated antioxidant strategies towards integrative, network-oriented approaches in the study of optic neuropathy.

## 1. Introduction

The optic nerve represents the principal afferent pathway of the visual system, providing the transmission of visual information from retinal ganglion cells (RGCs) to higher visual centers. Approximately 1.2 million axons originating from RGCs converge at the optic disc to form the optic nerve, rendering its structural and functional integrity critically important for vision [1]. Damage to the optic nerve, optic chiasm, or retrochiasmal visual pathways results in characteristic visual field defects and constitutes one of the leading causes of irreversible vision loss [2]. The clinical presentation of such damage is often nonspecific at early stages, underscoring the need for a deeper understanding of the underlying molecular mechanisms and for the development of targeted neuroprotective strategies. Retinal ganglion cell axons are particularly vulnerable to metabolic and oxidative stress due to their high energy demands, long unmyelinated intraocular segments, and strong dependence on mitochondrial function for the maintenance of axonal transport and electrophysiological activity [3,4]. Accumulating experimental and clinical data indicates that oxidative stress, mitochondrial dysfunction, and lipid peroxidation play a central role in the pathogenesis of various optic neuropathies, including glaucoma, ischemic optic neuropathy, and inflammatory or metabolic retinal disorders [3,4,5]. Disruption of redox homeostasis leads to excessive generation of reactive oxygen species (ROS), impaired mitochondrial function, and progressive degeneration of RGCs.

In recent years, ferroptosis—an iron-dependent form of regulated cell death characterized by the accumulation of lipid peroxides—has been increasingly recognized as a potential contributor to cellular injury in ocular diseases [6]. Dysregulation of antioxidant defense systems, including glutathione peroxidase 4 (GPX4) and other redox enzymes, may promote lipid peroxidation and neuronal vulnerability, further linking ferroptotic mechanisms to retinal ganglion cell degeneration [7,8].

Glaucoma is the leading cause of irreversible blindness worldwide, affecting more than 70 million individuals, and its prevalence is expected to increase over the coming decades [9]. Although reduction in intraocular pressure remains the primary therapeutic strategy, in a substantial proportion of patients the progression of optic nerve damage continues despite adequate pressure control [10,11,12,13]. This observation clearly indicates the existence of pressure-independent mechanisms of neurodegeneration and highlights the need for adjunctive neuroprotective therapies targeting oxidative stress, inflammation, and mitochondrial dysfunction. Although several mechanisms discussed in this context are shared across different optic neuropathies, the present study focuses primarily on glaucomatous optic neuropathy as the most prevalent form of progressive optic nerve degeneration.

Nutraceuticals are bioactive compounds of nutritional origin that have been attracting increasing attention as potential adjunctive agents for neuroprotection in optic nerve disorders. A number of nutraceuticals, including astaxanthin, α-lipoic acid (ALA), anthocyanins, omega-3 fatty acids (docosahexaenoic acid (DHA)/eicosapentaenoic acid (EPA)), coenzyme Q10 (CoQ10), and N-acetylcysteine (NAC), exhibit antioxidant, anti-inflammatory, and mitochondria-stabilizing properties that are directly relevant to retinal ganglion cell survival [14,15,16]. Preclinical and clinical studies support their potential: ALA has demonstrated a neuroprotective effect in experimental glaucoma and optic neuritis [17,18]; anthocyanins derived from blackcurrant have been associated with improvement of visual field parameters in patients with open-angle glaucoma [19]; coenzyme Q10 (CoQ10) stabilizes mitochondrial function and reduces oxidative damage in glaucomatous neurodegeneration [20,21]; and NAC attenuates oxidative stress and cellular loss in experimental models [22]. In addition, omega-3 fatty acids and citicoline-containing formulations have been associated with preservation of visual function in glaucoma [16]. Despite these extensive results, the molecular mechanisms through which nutraceuticals exert their neuroprotective effects on the optic nerve remain incompletely understood. Limited bioavailability, together with the complex role of inflammation, lipid metabolism, and oxidative stress in optic neuropathy, substantially complicates the mechanistic interpretation of the effects of these compounds [23,24,25,26]. Moreover, most available studies focus on individual nutraceuticals, whereas optic nerve pathology arises from the interaction of multiple unregulated pathways, including redox signaling, lipid metabolism, inflammatory cascades, and cell death programs. In particular, it remains unclear whether these compounds act through isolated antioxidant mechanisms or converge on shared regulatory modules associated with retinal ganglion cell vulnerability. Despite the growing interest and widespread use of nutraceuticals as potential neuroprotective agents in glaucoma, most studies have focused on individual compounds and their specific biological effects. Comparative analyses examining whether different nutraceuticals converge on shared molecular targets or regulatory pathways related to optic nerve degeneration remain limited. In this context, network pharmacology approaches may offer a systems-level perspective for identifying common functional modules associated with retinal ganglion cell vulnerability and neuroprotection. Such integrative in silico approaches provide an opportunity for systematic investigation of potential molecular targets and functional interactions of nutraceuticals, enabling the identification of convergent regulatory modules implicated in the pathophysiology of glaucomatous optic nerve degeneration. The primary research question addressed in this study is whether structurally diverse nutraceutical compounds converge on shared molecular targets and regulatory pathways relevant to glaucomatous optic nerve degeneration. To our knowledge, a systematic comparative network pharmacology analysis examining multiple nutraceutical compounds in the context of glaucomatous optic nerve degeneration has not been comprehensively performed. The aim of the present study was to investigate the potential molecular mechanisms through which selected nutraceutical compounds may exert neuroprotective effects on the optic nerve, using a systems-level in silico approach based on target prediction, protein–protein interaction analysis, and functional enrichment of biological pathways. The study sought to identify potential protein targets of selected nutraceuticals relevant to the retina and optic nerve; analyze the relationships among these targets through the construction of a protein–protein interaction network; and determine key molecular pathways and biological processes upon which these nutraceuticals exert convergent effects. The working hypothesis of the study is that nutraceuticals with established antioxidant and mitochondria-protective potential exert their neuroprotective effects not through single targets, but through coordinated modulation of interconnected molecular networks, involving redox homeostasis, lipid metabolism, inflammatory signaling, and programs of regulated cell death. Network pharmacological analysis is expected to reveal shared regulatory nodes and pathway modules that may be prioritized as potential therapeutic targets for future experimental and clinical studies.

## 2. Materials and Methods

### 2.1. Selection of Nutraceutical Compounds

For the purposes of the present in silico study, nutraceutical compounds with preclinical and/or clinical data of neuroprotective effects in diseases of the optic nerve or retina were selected. The compounds were selected based on published data for antioxidant activity, modulation of mitochondrial function, and effects on inflammatory processes. The following nutraceuticals were included in the analysis: astaxanthin, α-lipoic acid (ALA), cyanidin 3-glucoside, epigallocatechin gallate (EGCG), docosahexaenoic acid (DHA) and eicosapentaenoic acid (EPA), coenzyme Q10 (CoQ10), and N-acetylcysteine (NAC). The investigated compounds were represented through their SMILES formulas, which were used as input data for in silico prediction of molecular targets. The molecular formulas and corresponding SMILES of all analyzed compounds are summarized in Table 1.

### 2.2. Protein Target Prediction

Potential protein targets of each compound were identified using the SwissTargetPrediction (Swiss Institute of Bioinformatics, Lausanne, Switzerland) [27] platform, with Homo sapiens selected as the reference organism. To standardize the analysis and avoid imposing prior biological constraints, the top 15 highest-ranked molecular targets predicted via SwissTargetPrediction were used for each investigated compound. This threshold was selected to focus on the highest-confidence predicted interactions while avoiding the inclusion of lower-probability targets that may introduce noise into subsequent network analyses. Similar cut-off strategies using top-ranked predicted targets have been widely applied in network pharmacology studies to maintain interpretability of interaction networks. We acknowledge that alternative cut-offs could modify the size and density of the resulting networks; however, the selected threshold allows comparable and interpretable target sets across compounds while minimizing the inclusion of low-probability interactions. Functional enrichment analysis was performed using the STRING database (STRING Consortium, Zurich, Switzerland) [28]. Statistical significance of enrichment was assessed using the false discovery rate (FDR), calculated according to the Benjamini–Hochberg correction method for multiple testing. An FDR threshold of <0.05 was considered statistically significant. No prior tissue-specific selection (retinal- or optic nerve–restricted expression) was applied in the analysis, as the nutraceuticals act through systemic, multi-organ, and network-mediated mechanisms. This approach may introduce a degree of biological generalization; however, it allows the identification of systemic regulatory pathways that may indirectly influence retinal ganglion cell survival. The biological relevance to the optic nerve was assessed at the level of functional enrichment analysis and result interpretation, rather than through a priori exclusion of potentially relevant targets.

### 2.3. Protein–Protein Interaction Analysis

The list of protein targets was used to construct a protein–protein interaction (PPI) network using the STRING [28] database. The analysis was performed for Homo sapiens, applying a high-confidence interaction threshold (confidence score ≥ 0.700) and limiting the number of interactions per protein to a maximum of 20. The resulting network was analyzed with respect to key topological parameters, including the number of nodes, the number of edges, and the degree of connectivity of individual proteins. Highly connected proteins (hub proteins) were also identified, which may play a central regulatory role within the network and represent potential key targets for neuroprotection. Protein–protein interaction networks were constructed using the STRING database (version v12.0). The analysis was performed using the default STRING interaction sources, including experimental evidence, curated databases, gene co-expression, and text-mining-derived associations. All interaction sources contributed to the combined confidence score as implemented in the STRING platform, without additional weighting.

### 2.4. Functional Enrichment and Pathway Analysis

Functional enrichment of the molecular targets was performed using the built-in tools available in STRING, which integrate information from established biological databases, including Gene Ontology, KEGG, Reactome and WikiPathways. Statistically significant enriched pathways were identified based on an FDR-adjusted *p*-value threshold of <0.05, automatically calculated by the STRING database. Clustering of functionally related pathways was conducted using the clustering analysis tools integrated in STRING, allowing the identification of convergent functional modules for subsequent biological interpretation [28] (Table S1).

## 3. Results

Based on the analytical methodology described above, predicted molecular targets and functional interactions were identified that together form potential regulatory pathways for the actions of the investigated compounds. The initial analysis of the predicted molecular targets revealed that the different nutraceuticals interact with partially overlapping sets of proteins, including enzymes, nuclear receptors, and regulatory proteins. From a biological perspective, the most relevant targets are presented in Table 2, which serves as a framework for the subsequent analysis of individual compounds and allows rapid visual assessment of the predominant functional pathways of individual nutraceuticals.

To facilitate comparison between the analyzed compounds, the main topological parameters of the protein–protein interaction networks are summarized in Table 3.

To link the predicted molecular targets with existing biological and clinical data, Table 4 summarizes key proteins identified through the in silico analysis, together with information on their tissue expression, disease associations, and functional classification, derived from publicly available databases and literature sources. The presented information is intended to support the biological plausibility of the results, without providing direct experimental validation.

### 3.1. Astaxanthin

#### 3.1.1. Protein–Protein Interaction Analysis

**Figure 1:**
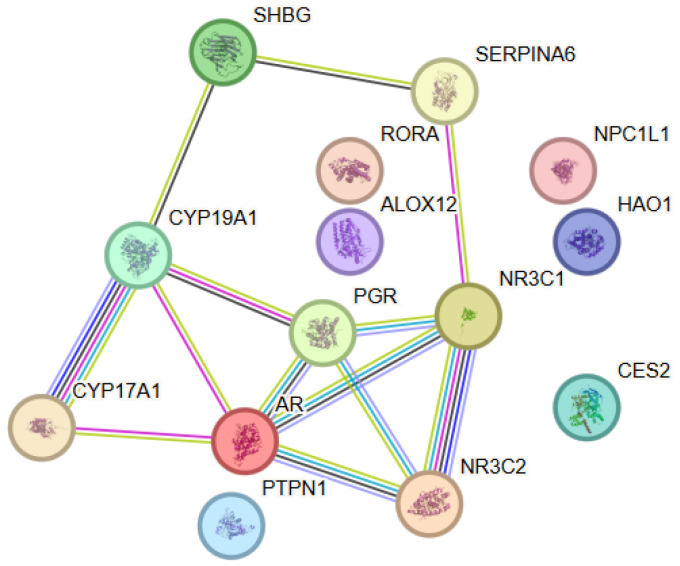
Protein–protein interaction (PPI) network constructed using STRING v12.0 for the selected astaxanthin-associated target proteins. The nodes represent proteins, while the edges represent functional associations between them. Nodes (circles) represent individual proteins, where color is used only for visual distinction and does not indicate biological categories, and each node corresponds to a single gene product (with splice variants merged); edges represent functional associations, with different colors indicating the type of supporting evidence (light blue for experimentally validated interactions, purple/pink for curated databases, green for gene co-expression, and yellow for text mining), while line thickness reflects the strength of the combined STRING confidence score, with thicker lines indicating higher-confidence associations.

The analysis aimed at elucidating the functional relationships associated with astaxanthin revealed a network comprising 14 proteins (nodes) and 13 interactions (edges), with the observed connectivity being significantly higher than that expected for a random set of proteins (PPI enrichment *p*-value = 4.44 × 10^−16^) (Figure 1). This finding suggested that the analyzed proteins were biologically related and participated in shared functional processes. Within the astaxanthin-associated PPI network, several proteins showed higher connectivity, including AR (degree = 5), followed by CYP19A1, NR3C1 and PGR (degree = 4), indicating their central role within the interaction network. The network was dominated by nuclear steroid receptors and enzymes involved in steroid metabolism, which together form a densely interconnected functional cluster. The strongest interactions were observed between CYP17A1 and CYP19A1, as well as between NR3C1 and NR3C2, reflecting coordinated regulation within steroidogenic and glucocorticoid signaling pathways. The androgen receptor (AR) exhibited multiple high-confidence connections with NR3C1, NR3C2, PGR, CYP17A1 и CYP19A1, indicating its central role within the network. The node degree analysis identified AR, NR3C1 and CYP19A1 as hub proteins with the highest number of interactions. Their topological positions suggested that they acted as key integrators of steroid signaling and metabolic processes within the investigated network.

#### 3.1.2. Functional Enrichment Analysis

The functional analysis using Gene Ontology revealed enrichment of biological processes associated with intracellular signaling mediated by steroid hormone receptors, glucocorticoid metabolism, and general steroid metabolism. Analysis of molecular functions showed a predominance of categories such as steroid binding, nuclear receptor activity, and interaction with transcriptional co-activators, supporting the central role of ligand-dependent transcriptional regulators. The pathway enrichment analysis performed using Reactome further supported the functional coherence of the network. Significantly enriched pathways included nuclear receptor-mediated transcription, SUMOylation of intracellular receptors, the HSP90 chaperone cycle associated with steroid hormone receptors, and steroid hormone metabolism. These pathways highlighted the importance of receptor activation, post-translational regulation, and chaperone-mediated stabilization in coordinating steroid-dependent cellular responses.

### 3.2. α-Lipoic Acid

#### 3.2.1. Protein–Protein Interaction Analysis

**Figure 2:**
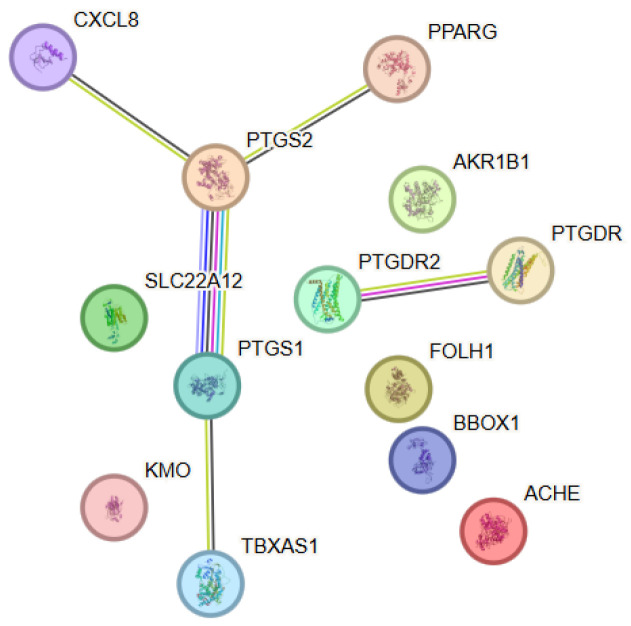
Protein–protein interaction (PPI) network constructed using STRING v12.0 for the selected target proteins associated with α-Lipoic acid. The nodes represent proteins, while the edges represent functional associations between them. Color coding and evidence types are described in Figure 1.

The investigation of the functional relationships among molecular targets associated with α-lipoic acid revealed, through protein–protein interaction (PPI) analysis, a network comprising 13 proteins (nodes) connected by various functional interactions (edges), forming a structured biological model (Figure 2). The network architecture revealed a central cluster predominated by enzymes involved in arachidonic acid metabolism and prostanoid signaling. Key node elements within the network included PTGS1 (COX-1) and PTGS2 (COX-2), which exhibited a high degree of connectivity and served as functional cores. These enzymes were connected with both eicosanoid-metabolizing enzymes and receptors and regulatory proteins associated with inflammatory processes. Notable interactions were observed between PTGS2 and CXCL8, as well as between PTGS1 and TBXAS1, reflecting coordinated regulation of inflammatory and platelet signaling pathways. The presence of PTGDR and PTGDR2 highlighted the involvement of prostaglandin receptors within the network and linked enzymatic activity to receptor-mediated cellular responses. Additional peripheral yet functionally relevant nodes included PPARG, AKR1B1, FOLH1, BBOX1 and ACHE, which extended the network towards metabolic regulation, redox homeostasis, and neurotransmitter-related activity. Although these proteins lacked direct connectivity within the network, they contributed to its functional heterogeneity. The node degree analysis identified PTGS1 and PTGS2 as the principal hub proteins. Their central topological positions suggested a key integrative role between inflammatory signaling, lipid metabolism, and cellular protective mechanisms associated with the action of α-lipoic acid. Within the α-lipoic acid–associated PPI network, PTGS2 (degree = 3) showed the highest connectivity, followed by PTGS1 (degree = 2), while several other proteins such as CXCL8, PPARG, PTGDR, PTGDR2 and TBXAS1 exhibited lower connectivity (degree = 1).

#### 3.2.2. Functional Enrichment Analysis

The functional analysis revealed enrichment of biological processes associated with prostanoid metabolism, the inflammatory response, lipid signaling, and oxidation-reduction processes. Within the molecular functions, predominant categories were associated with enzymatic activity acting on polyunsaturated fatty acids, prostaglandin synthesis, and receptor-mediated signaling. Pathway analysis indicated convergence towards pathways linked to the cyclooxygenase cascade, regulation of inflammation, and interactions between metabolic and signaling mechanisms. These results highlight the role of α-lipoic acid as a molecule affecting key nodes within inflammatory and metabolic regulation, without being restricted to a single target.

### 3.3. Cyanidin-3-Glucoside

#### 3.3.1. Protein–Protein Interaction Analysis

**Figure 3:**
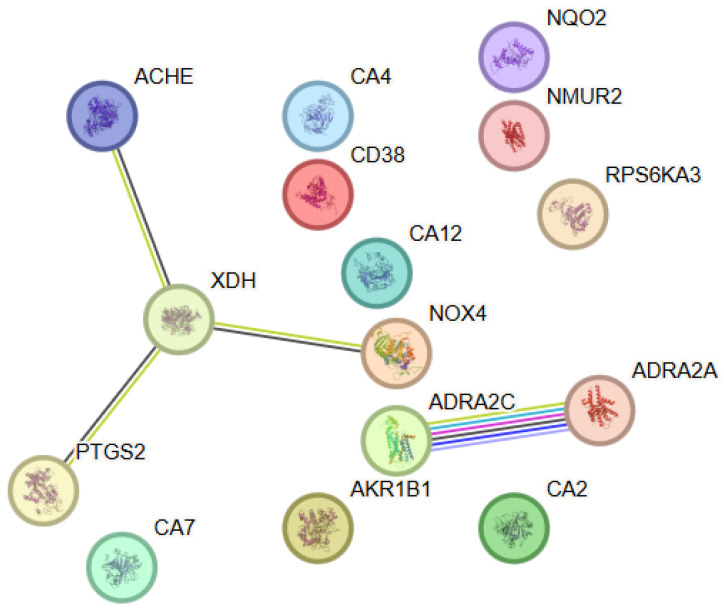
Protein–protein interaction (PPI) network constructed using STRING v12.0 for the selected target proteins associated with Cyanidin-3-clucoside. The nodes represent proteins, while the edges represent functional associations between them. Color coding and evidence types are described in Figure 1.

The analysis conducted to elucidate the functional relationships among the identified molecular targets of cyanidin-3-glucoside (C3G) revealed a network comprising 15 proteins (nodes) and a limited number of functional interactions (edges) (Figure 3). Despite the moderate network density, the observed connectivity exceeded that expected for a random set of proteins, indicating the presence of a functionally organized biological module. Within this PPI network, XDH showed the highest connectivity (degree = 3), whereas several other proteins, including ACHE, ADRA2A, ADRA2C, NOX4 and PTGS2, exhibited lower connectivity (degree = 1). The network was characterized by a distinct functional axis dominated by enzymes and regulatory proteins involved in redox homeostasis, ROS metabolism, and inflammatory signaling. Prominent central nodes included PTGS2 (COX-2), NOX4, XDH and ACHE, which formed a core associated with the regulation of oxidative and inflammatory stress. PTGS2 established functional associations with several metabolic and regulatory proteins, underscoring its role as an integrator of inflammatory signals dependent on eicosanoid metabolism. Additional nodes, such as AKR1B1 and NQO2, extended the network towards pathways associated with detoxification and redox adaptation, while the involvement of carbonic anhydrases and adrenergic receptors suggested potential relevance to metabolic and vascular regulation.

#### 3.3.2. Functional Enrichment Analysis

The functional analysis using Gene Ontology revealed significant enrichment of biological processes associated with the regulation of oxidative stress, inflammatory response, and lipid mediator metabolism. Among the dominant categories, prominent processes were associated with eicosanoid metabolism, cellular defense responses against oxidative damage, and redox-dependent signaling. Molecular function analysis indicated enrichment of categories encompassing oxidoreductase activity, enzymatic regulation of ROS, and involvement in inflammation-mediated pathways. Pathway enrichment analysis further supported the involvement of C3G in pathways associated with COX-2-mediated inflammatory signaling, redox adaptation, and cellular responses to metabolic stress, consistent with its reported antioxidant and anti-inflammatory profile.

### 3.4. Epigallocatechin-3-Gallate

#### 3.4.1. Protein–Protein Interaction Analysis

**Figure 4:**
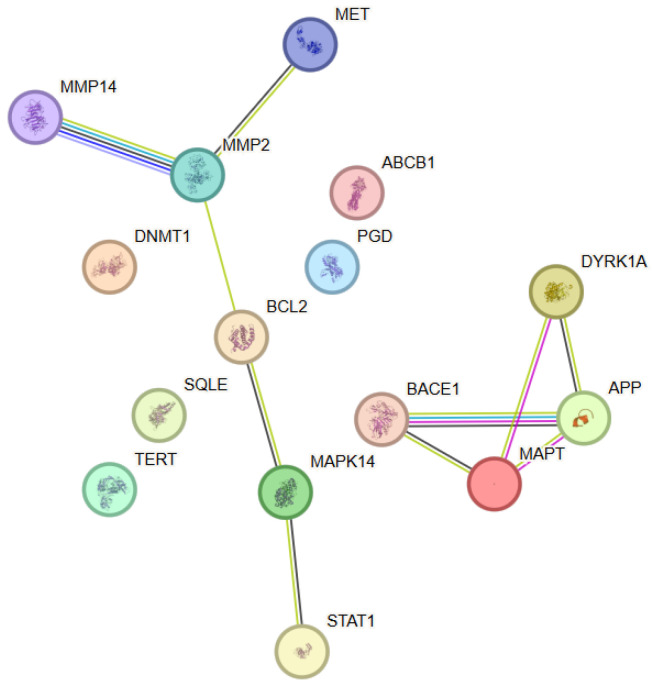
Protein–protein interaction (PPI) network constructed using STRING v12.0 for the selected target proteins associated with Epigallocatechin-3-gallate. The nodes represent proteins, while the edges represent functional associations between them. Color coding and evidence types are described in Figure 1.

The analysis of the functional relationships among the molecular targets of epigallocatechin-3-gallate (EGCG) revealed a network comprising 15 proteins (nodes) connected by multiple functional associations, with the observed connectivity being significantly higher than that expected for a random set of proteins (Figure 4). This indicated the presence of a biologically coherent protein module. Within this network, APP, MAPT and MMP2 showed the highest connectivity (degree = 3), while several additional proteins, including BACE1, BCL2, DYRK1A and MAPK14, displayed moderate connectivity (degree = 2). The network architecture was characterized by distinct clusters associated with proteins involved in signal transduction, regulation of cellular stress, and control of apoptosis and cell survival. Central nodes within the network included MAPK14 (p38α), BCL2, and STAT1, which formed a functional axis linking stress-activated signaling, transcriptional regulation, and epigenetic control. An additional cluster, comprising BACE1, APP, MAPT and DYRK1A, connected EGCG with pathways associated with proteolysis, neuronal stability, and protein homeostasis. Node degree analysis identified MAPK14 and BCL2 as nodes of high topological significance, which suggested their key role in the integration of signals related to the anti-inflammatory, antioxidant, and cytoprotective effects of EGCG.

#### 3.4.2. Functional Enrichment Analysis

The functional enrichment analysis using Gene Ontology revealed significant enrichment of biological processes associated with cellular stress response, regulation of apoptosis, signaling via MAPK cascades, and epigenetic control of transcription. These processes were highly consistent with the well-documented antioxidant and anti-inflammatory properties of EGCG. Molecular function analysis indicated predominance of categories involving protein kinase activity, regulation of transcription factors, DNA methylation, and control of cell survival. Pathway enrichment analysis further corroborated the involvement of EGCG in pathways associated with stress-activated signaling, apoptotic regulation, and neuronal protection, underscoring its potential role as a multi-target modulator of cellular adaptive mechanisms.

### 3.5. Docosahexaenoic Acid

#### 3.5.1. Protein–Protein Interaction (PPI) Analysis

**Figure 5:**
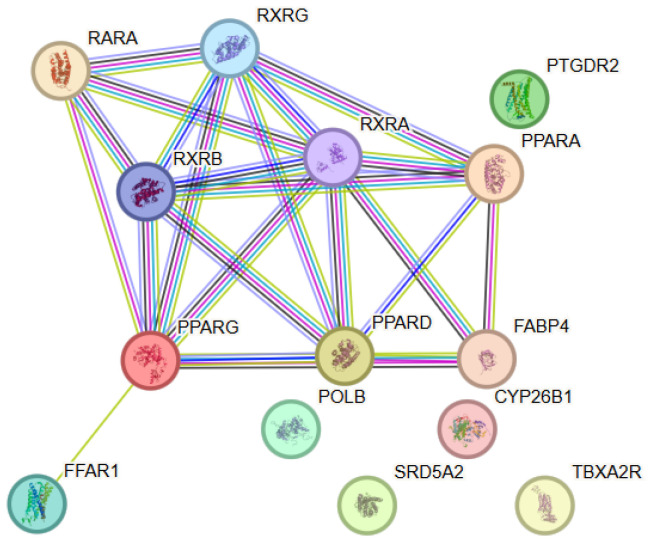
Protein–protein interaction (PPI) network constructed using STRING v12.0 for the selected target proteins associated with Docosahexaenoic acid. The nodes represent proteins, while the edges represent functional associations between them. Color coding and evidence types are described in Figure 1.

To elucidate the functional relationships among the identified molecular targets of docosahexaenoic acid, a network comprising 14 proteins (nodes) and a substantial number of functional interactions (edges) was obtained. Within this PPI network, PPARG and RXRA exhibited the highest connectivity (degree = 7), followed by PPARD, RXRB and RXRG (degree = 6) and PPARA (degree = 5), indicating a central role of nuclear receptor signaling components within the interaction network (Figure 5). The observed network connectivity was significantly higher than that expected for a random set of proteins (PPI enrichment *p* < 0.001), indicating that the included proteins were biologically related and participated in shared regulatory and metabolic processes. The network was characterized by a predominance of proteins involved in lipid metabolism, anti-inflammatory signaling, regulation of oxidative stress, and neuronal homeostasis. The identified interactions formed a functionally integrated module associated with membrane stability, mitochondrial function, and cellular protection. Node degree analysis identified several hub proteins exhibiting an increased number of interactions and occupying central topological positions within the network. Their role suggested a potential role in the coordinated regulation of lipid-dependent signaling cascades and cellular adaptive mechanisms.

#### 3.5.2. Functional Enrichment Analysis

The functional enrichment analysis revealed significant enrichment of biological processes associated with lipid metabolism, regulation of the inflammatory response, oxidative stress, and cellular survival. Among the most prominently represented categories were processes associated with fatty acid metabolism, membrane organization, and lipid mediator-dependent signaling. Analysis of molecular functions showed enrichment of categories including lipid binding, receptor activity, and activity of enzymes involved in redox regulation, as well as interactions with proteins regulating transcription and cellular stress responses.

### 3.6. Eicosapentaenoic Acid

#### 3.6.1. Protein–Protein Interaction (PPI) Analysis

**Figure 6:**
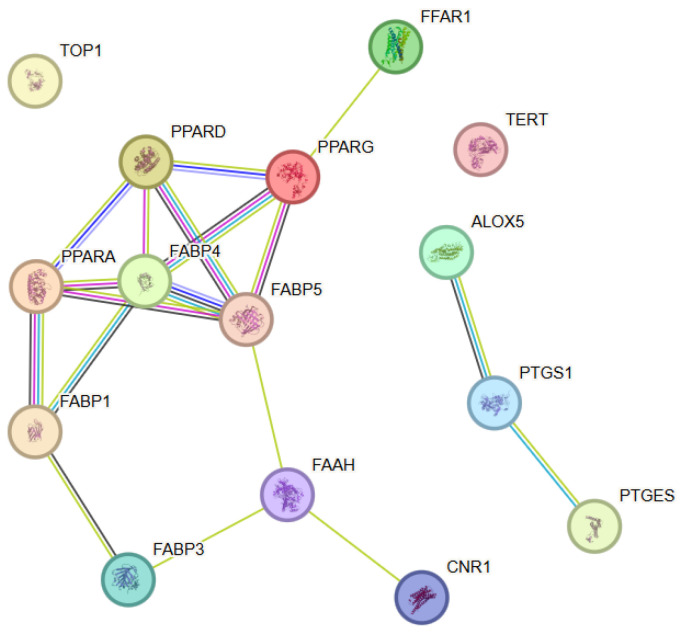
Protein–protein interaction (PPI) network constructed using STRING v12.0 for the selected target proteins associated with Eicosapentaenoic acid. The nodes represent proteins, while the edges represent functional associations between them. Color coding and evidence types are described in Figure 1.

To elucidate the functional relationships among molecular targets associated with eicosapentaenoic acid (EPA), the protein–protein interaction analysis performed resulted in the construction of a network comprising 15 proteins (nodes) and multiple statistically significant interactions (edges) (Figure 6). Within this PPI network, FABP4 and FABP5 showed the highest connectivity (degree = 5), followed by PPARA, PPARD and PPARG (degree = 4), indicating their central role within the interaction network. The observed network density was significantly higher than that expected for a random set of proteins, corroborating the presence of a functionally connected biological module. The network was characterized by a distinct organization around proteins involved in lipid metabolism, inflammatory signaling, and regulation of oxidative stress. Dense connections were observed between enzymes and regulatory proteins involved in polyunsaturated fatty acid metabolism, eicosanoid signaling, and nuclear receptor–mediated transcriptional regulation. The node degree analysis identified several hub proteins occupying central topological positions within the network and functioning as integrators of metabolic and signaling pathways. These proteins displayed a high number of interactions and likely played a key role in the coordination of anti-inflammatory and neuroprotective effects associated with EPA.

#### 3.6.2. Functional Enrichment Analysis

The functional enrichment analysis revealed significant enrichment of biological processes associated with lipid metabolism, regulation of the inflammatory response, cellular homeostasis, and responses to oxidative stress. These results highlighted the central role of EPA in the modulation of inflammation-mediated cellular processes and the maintenance of membrane and mitochondrial stability. Analysis of molecular functions showed predominance of categories including lipid binding, enzymatic activity associated with fatty acid metabolism, and regulatory functions related to signal transduction. These functions reflected the ability of EPA to influence key enzymatic and regulatory nodes within cellular metabolism.

### 3.7. Coenzyme Q10

#### 3.7.1. Protein–Protein Interaction (PPI) Analysis

**Figure 7:**
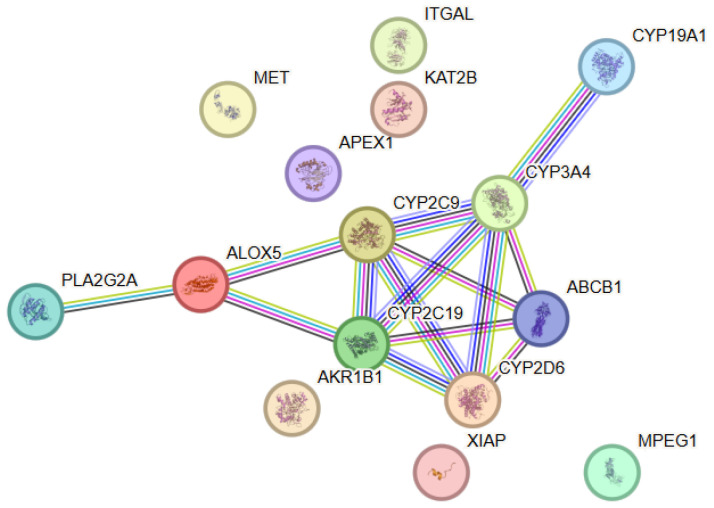
Protein–protein interaction (PPI) network constructed using STRING v12.0 for the selected target proteins associated with CoenzymeQ10. The nodes represent proteins, while the edges represent functional associations between them. Color coding and evidence types are described in Figure 1.

The protein–protein interaction (PPI) analysis associated with coenzyme Q10 revealed a network comprising 15 proteins (nodes) and a statistically significant number of interactions (edges), with the observed connectivity being significantly higher than that expected for a random set of proteins, which corroborates the presence of a functionally organized biological module (Figure 7). Within this PPI network, CYP2C19, CYP2C9 and CYP3A4 exhibited the highest connectivity (degree = 5), followed by ABCB1 and CYP2D6 (degree = 4), indicating their central role within the interaction network. The network was characterized by a pronounced concentration of proteins involved in mitochondrial energy metabolism, redox homeostasis, and cellular protection against oxidative stress. Dense functional connections were observed between enzymes and regulatory proteins associated with mitochondrial function and antioxidant defense. The node degree analysis identified several hub proteins occupying central topological positions within the network. These proteins exhibited a high number of interactions and likely functioned as key integrators of mitochondrial energy production and CoQ10-mediated cellular protective mechanisms.

#### 3.7.2. Functional Enrichment Analysis

The functional enrichment analysis revealed significant enrichment of biological processes associated with mitochondrial respiration, cellular energy metabolism, regulation of oxidative stress, and maintenance of redox balance. These results highlighted the central role of CoQ10 in maintaining mitochondrial function and cellular viability. Analysis of molecular functions showed predominance of categories including electron transport, oxidoreductase activity, coenzyme binding, and regulatory functions associated with the mitochondrial membrane. These functional characteristics reflect the key involvement of CoQ10 in the electron transport chain and antioxidant defense.

### 3.8. N-Acetylcysteine

#### 3.8.1. Protein–Protein Interaction (PPI) Analysis

**Figure 8:**
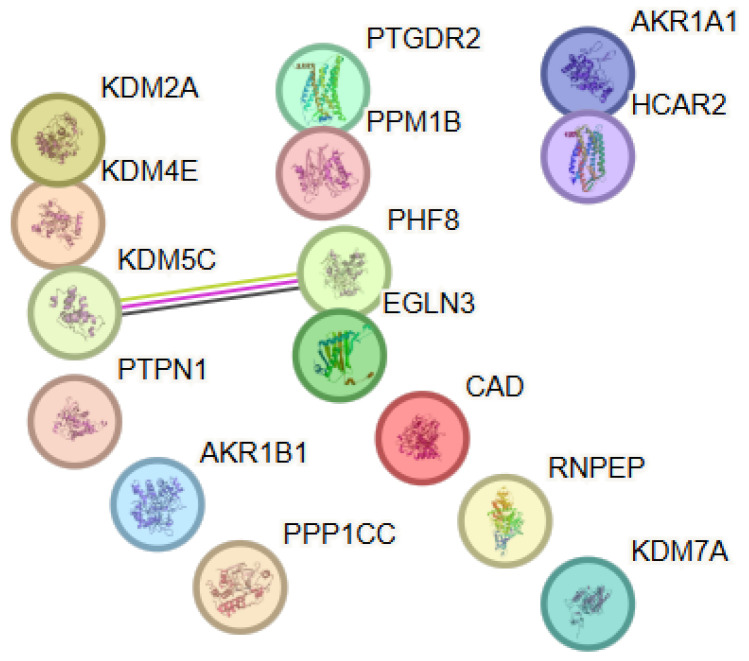
Protein–protein interaction (PPI) network constructed using STRING v12.0 for the selected target proteins associated with N-acetylcysteine. Color coding and evidence types are described in Figure 1.

The predicted targets of NAC formed a sparsely connected network with a non-significant PPI enrichment *p*-value (0.147), indicating that the observed connectivity does not exceed random expectation. Therefore, the interpretation of this network is primarily based on functional enrichment rather than network topology (Figure 8). Notably, several targets are involved in histone lysine demethylation and related epigenetic regulatory processes. These proteins share functional roles but are not necessarily expected to form dense protein–protein interaction networks, as they often act within chromatin-associated complexes or through indirect regulatory mechanisms. Accordingly, the observed functional grouping likely reflects shared biological roles rather than direct physical interactions between the proteins. Within this PPI network, KDM5C and PHF8 showed the highest connectivity (degree = 1), while the remaining proteins exhibited minimal or no direct interactions within the network. The network was characterized by a distinct functional organization around proteins involved in epigenetic regulation, particularly the processes of histone lysine demethylation and the enzymatic activity of 2-oxoglutarate–dependent dioxygenases. Functional grouping of enzymes and regulatory proteins engaged in chromatin modification and control of transcriptional activity was observed, despite the weak direct protein–protein connectivity. Node degree analysis showed a low average node degree of 0.133 and an average local clustering coefficient of 0.133, reflecting the absence of clearly defined hub proteins and suggesting that NAC-associated effects were mediated through modulation of functionally related but weakly interacting molecular targets. These observations suggest that functional convergence among NAC-associated targets may arise from shared biochemical roles rather than dense protein–protein interaction connectivity.

#### 3.8.2. Functional Enrichment Analysis

**Figure 9:**
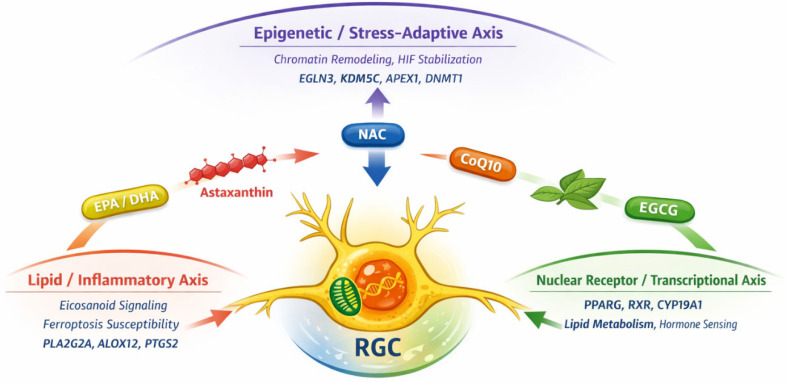
Conceptual framework illustrating the convergent molecular pathways modulated by the investigated nutraceuticals in optic nerve neuroprotection. The conceptual illustration presented in this figure was created with the assistance of AI-based image generation tools.

The enrichment results presented here are interpreted in a descriptive manner, focusing on functional patterns rather than formal statistical comparison between networks. The functional enrichment analysis revealed significant enrichment of biological processes associated with histone lysine demethylation, including demethylation of H3-K36, H3-K9, and H4-K20. These results highlighted the central role of epigenetic mechanisms in the functional profile of the identified targets and pointed to the involvement of NAC in the modulation of chromatin structure and transcriptional regulation. Analysis of molecular functions showed predominance of categories including 2-oxoglutarate–dependent dioxygenase activity, histone demethylase activity, and oxidoreductase functions associated with metal (iron) binding. These functions reflected the enzymatic profile of proteins belonging to the Jumonji family of histone demethylases, which occupy a central position in the regulation of epigenetic modifications. To facilitate integrative interpretation of the predicted targets and enriched pathways, the main findings are summarized in a conceptual framework (Figure 9).

## 4. Discussion

The novelty of the present work lies primarily in the comparative systems-level analysis of multiple nutraceutical compounds and the identification of convergent functional pathways rather than in the experimental validation of specific molecular mechanisms. In the context of the present study, convergence refers to the identification of shared functional pathways, regulatory modules, or highly connected molecular nodes that appear across multiple nutraceutical-associated networks. Such convergence may manifest as overlapping enriched pathways, common hub proteins, or functionally related molecular targets involved in similar biological processes. Glaucoma and related optic neuropathies remain among the leading causes of irreversible vision loss worldwide. Their clinical and societal burden continues to increase in parallel with global population aging. Worldwide data from 2020 report that tens of millions of individuals are affected by glaucoma, with projections indicating a further increase by 2040 [134]. Despite the proven efficacy of therapeutic approaches connected with lowering intraocular pressure (IOP), both clinical experience and evidence from the literature support the existence of IOP-independent mechanisms of disease progression. These mechanisms include mitochondrial dysfunction, oxidative stress, neuroinflammation, dysregulation of lipid signaling, and activation of regulated cell death programs [13,135]. In the context of these facts, nutraceuticals are not considered alternatives to standard therapies but rather potential adjunctive modulators simultaneously acting on multiple pathogenetic pathways, which represents a key strategy for diseases with complex, network-based pathogenesis [136].

### 4.1. Convergence of Target Pathways and Their Biological Relevance

In the present in silico study, the main outcome was not limited to an isolated list of predicted molecular targets but rather lay in the observed convergence of different nutraceutical compounds toward a limited number of functional domains associated with the susceptibility of RGCs and their axons to injury. These domains included inflammatory cascades and eicosanoid metabolism, redox regulation and mitochondrial homeostasis, nuclear receptor—and transcription-mediated regulation, as well as epigenetically active enzymatic components. Such functional orientation/convergence is consistent with the contemporary concept of “network pharmacology”, according to which therapeutic effects are often achieved through subtle modulation of several key nodes rather than through intervention aimed at one dominant target. For some of the analyzed nutraceuticals, a more pronounced involvement of inflammation-lipid pathways was observed, including prostanoid and eicosanoid signaling mediated by specific enzymatic and receptor nodes. However, the extent and mechanisms of involvement of these pathways varied substantially among individual compounds and did not represent a universal mechanism of action. The biological relevance of this convergence was determined, on the one hand, by the role of lipid mediators in the regulation of processes associated with progressive optic nerve dysfunction, and, on the other hand, by the growing interest in omega-3 and other lipid-based supplements, as well as bioactive compounds with combined anti-inflammatory and antioxidant profiles, in the management of glaucoma [137]. Accordingly, the in silico approach considered inflammatory processes not as a universal mechanism but as functionally organized processes around specific molecular nodes. Network analysis enabled the identification of such nodes and created opportunities for favorable results from subsequent experimental investigations, for example through the measurement of COX-2 and prostanoid metabolites, markers of glial activation, or downstream transcriptional responses.

Another predominant pathway identified for some of the analyzed nutraceuticals was related to mitochondrial redox regulation, bioenergetic balance, and metabolic adaptation. It is biologically plausible that RGCs, due to their high energy demands, are particularly susceptible to mitochondrial dysfunction, increased oxidative stress, and impaired axonal transport. To our knowledge, a comparative systems-level analysis of groups of antioxidant nutraceuticals in the context of glaucomatous optic neuropathy has not yet been conducted. Most available studies have examined individual compounds separately, without assessing potential mechanistic convergence within shared molecular networks. An integrative network-based approach may therefore provide additional insight into common regulatory pathways implicated in the pathophysiology of glaucoma. In this context, coenzyme Q10 (CoQ10) is frequently discussed in both experimental and clinical literature as a mitochondria-relevant antioxidant, while nutraceutical interventions in general are considered supportive neuroprotective strategies in the management of glaucoma [138]. It should be emphasized that the network analysis performed in the present study did not imply direct modulation of the components of the electron transport chain, but rather pointed to effects on redox and metabolic nodes that influenced mitochondrial functional stability. Mitochondrial pathways may be viewed as a mechanistic bridge between the susceptibility of RGCs to injury and potential therapeutic strategies, allowing the results of the present analysis to be interpreted as a hypothesis for subsequent experimental investigations rather than as definitive evidence.

In recent years, ferroptosis has become recognized as a potentially responsible mechanism for ocular and retinal injury, in which iron metabolism and the accumulation of lipid peroxides play a central role [139]. In the present study, this connection was considered solely as a conceptual analogy, as several of the identified functional axes—lipid mediators, redox regulation, and mitochondrial dysfunction—intersected at the level of lipid peroxidation, without asserting a direct involvement of ferroptosis.

In contrast to the other nutraceuticals, network analysis for NAC revealed a very weak PPI signal (15 nodes, 1 edge; PPI enrichment *p*-value ≈ 0.147), which did not support the presence of a densely interacting protein module. However, this result should not be interpreted as an absence of biological activity. Rather, the predicted targets likely represented functionally related but not directly interacting proteins, such as enzymes belonging to the same catalytic family. Consistent with this observation, the enrichment profile for NAC was strongly focused on histone demethylation and 2-oxoglutarate–dependent dioxygenases (JmjC enzymes), representing a typical example of biologically meaningful activity in the absence of pronounced PPI network organization. This interpretation is consistent with published data showing that NAC and its derivatives (e.g., N-acetylcysteine amide) exert cytoprotective effects against oxidative stress and mitochondrial damage in cellular models [140]. It should be noted that the NAC-associated network did not demonstrate statistically significant PPI enrichment. Therefore, the biological interpretation in this case relies primarily on functional enrichment patterns rather than on network topology. Histone demethylases and other epigenetic regulators often participate in shared metabolic or chromatin-modifying pathways without necessarily forming dense direct protein–protein interaction networks.

### 4.2. Clinical Evidence on Nutraceuticals Versus the Literature

Neuroprotective strategies in glaucoma are complex with respect to their translation from preclinical models to real-world clinical efficacy. This is particularly true for nutraceuticals, which are characterized by substantial heterogeneity in terms of bioavailability, dosing regimens, combinations, and ultimate biological effect. Despite these limitations, a growing body of review articles and clinical studies has examined compounds such as citicoline, omega-3 fatty acids, and other bioactive molecules as potential adjunctive agents, with clinical investigations in this area continuing to expand [141]. In this context, the present work offers a conceptual framework for rational selection and combination of nutraceutical compounds based on their predicted network profiles. For example, the combination of a nutraceutical targeting prostanoid-inflammatory pathways with another affecting mitochondrial and redox regulation could theoretically provide broader coverage of diverse pathogenetic components in comparison with random or empirical supplementation.

### 4.3. Limitations of the In Silico Approach

The present study has several inherent limitations. The predicted targets are probabilistic and based on chemical similarity (SwissTargetPrediction), and therefore require experimental validation [142]. The STRING PPI analysis reflects currently available functional associations, and small target sets may not yield statistically enriched networks, as observed for NAC. Enrichment results are dependent on selected statistical thresholds and correction methods, which should be considered when interpreting pathway associations. In addition, pharmacokinetic and tissue-specific factors—including retinal penetration, administered doses, and active metabolites—are not incorporated into the present model and may influence biological relevance. Future studies should therefore combine target-oriented molecular validation with functional assays in retinal ganglion cell models to confirm the mechanistic implications suggested by this analysis [139].

### 4.4. Interconnections Between Mechanisms of Multimodal Neuroprotection

**Figure 10:**
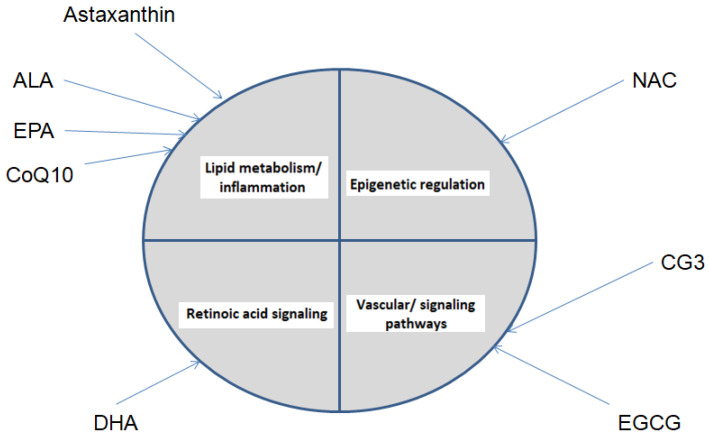
Mechanistic convergence of nutraceutical compounds across shared biological pathways.

The results of the present in silico study indicate that, despite the pronounced chemical and functional heterogeneity of the examined nutraceutical compounds, their predicted molecular targets are not randomly distributed. Instead, they show a tendency to converge on a limited number of biological pathways linked to key pathophysiological mechanisms of optic neuropathy. In the context of this study, convergence is defined as the recurrence of shared enriched biological pathways and functional processes across different compounds, rather than overlap at the level of individual molecular targets or identical network topology. This particular convergence constitutes the central conceptual message of the study. Among the identified functional axes, the inflammatory–lipid axis, represented by enzymes and receptors involved in eicosanoid and prostanoid metabolism, stands out. This axis is predominant across several of the analyzed nutraceuticals and is of particular relevance in the context of glaucoma, in which chronic low-grade inflammation, glial activation, and local vascular and fibro-inflammatory dysregulation are considered key factors for the progressive loss of RGCs [143]. Within the present analysis, the inflammatory–lipid axis is most prominently represented for α-lipoic acid and eicosapentaenoic acid, whereas for other compounds it is only partially expressed or plays a secondary role relative to redox-, epigenetic-, or signaling-adaptive mechanisms. To facilitate comparison between compounds and to visually illustrate the concept of mechanistic convergence, an integrated summary diagram is presented (Figure 10).

Although several compounds converge on common biological pathways, particularly those related to lipid metabolism and inflammation, some compound-specific patterns can also be distinguished. For instance, DHA shows a stronger association with retinoic acid signaling, while NAC is mainly linked to epigenetic regulatory processes. In contrast, CG3 and EGCG are more closely related to signaling and vascular mechanisms. Taken together, these findings suggest that the analyzed nutraceuticals may exert complementary effects through both shared and distinct biological pathways.

At the same time, it is well established that many nutraceuticals exert their biological effects through modulation of inflammatory–lipid signaling pathways, supporting the network-mediated nature of the observed convergence [144]. These results suggest that lipid-inflammatory signaling represents a fundamental, but not exclusive, mechanism of action of nutraceuticals, which calls for consideration of additional layers of regulation. Network analysis enables anti-inflammatory effects to be interpreted not as diffuse and nonspecific, but as structured around specific molecular nodes, creating opportunities for a more precise experimental validation.

The second clearly defined axis was the mitochondrial–redox axis, which encompassed enzymes and regulatory factors associated with mitochondrial redox regulation, antioxidant defense, bioenergetic balance, and metabolic adaptation, without implying direct modulation of components of the electron transport chain. The susceptibility of RGCs to mitochondrial dysfunction is well documented in the literature, with existing data highlighting the central role of mitochondrial metabolism and oxidative stress in optic neuropathies. From a biological perspective, this lends strong support to the relevance of the observed orientation towards mitochondria-associated pathways. From a network-based perspective, the identified axis may be regarded as a functional scaffold through which nutraceuticals potentially enhance cellular resilience to metabolic and oxidative stress, without necessitating the complete blocking of a single signaling pathway [145,146]. This strategy is supported by findings of pronounced functional and spatial specialization of mitochondria in RGCs, suggesting opportunities for network-mediated regulation [147]. In addition to metabolic and inflammatory processes, the analysis also revealed an important role of epigenetic and stress-adaptive mechanisms, which potentially provide longer-term regulation of cellular responses. At a higher regulatory level, the identification of nuclear-receptor-related modules suggests that some of the effects of nutraceuticals may be mediated through transcriptional regulation, rather than solely through direct antioxidant activity.

The third axis was characterized by the identification of a module involving nuclear-receptor and transcriptional regulation, particularly via steroid and related receptors, which placed the analyzed nutraceuticals within the context of long-term adaptive regulation of cellular responses, rather than solely as agents with short-lived antioxidant activity. Activation of nuclear receptors has been shown to lead to a sustained change in transcriptional programs involved in the regulation of inflammation, metabolism, and cell survival. In this context, some of the observed effects could be achieved through persistent changes in the transcriptional profiles of cells, which is consistent with the established mechanisms of action of nuclear receptors [61,148]. N-acetylcysteine influences redox homeostasis and has been associated with modulation of epigenetic mechanisms, and it is assumed that alterations in oxidative status may affect transcriptional regulation and chromatin dynamics. Data from experimental and translational models indicates that NAC may participate in epigenetically mediated adaptive responses, particularly in the context of stress-related and hypoxic conditions, supporting the possibility of long-term effects on cellular function [149]. From a broader neurobiological perspective, epigenetic regulation is well recognized as playing a key role in the development and long-term adaptation of the nervous system, providing a conceptual framework for interpreting such effects beyond classical antioxidant mechanisms [150]. At the same time, it should be noted that antioxidant therapy with NAC does not lead to the prevention of epigenetic changes, underscoring the context-dependent nature of these mechanisms and the need for cautious interpretation [151]. Overall, the results support the concept that nutraceuticals may be viewed as multi-target modulators, acting on distinct yet complementary pathogenetic pathways. This has direct translational possibilities, since the rational combination of nutraceuticals with different network profiles could provide broader coverage of pathological mechanisms than a monotherapeutic approach (Table 5).

**Table 5:** Convergent and specific molecular axes of the analyzed nutraceuticals.

Nutraceutical	Predominant Molecular Axis	Key Targets and Functions (Representative)	Biological Relevance to the Optic Nerve
α-lipoic acid (ALA)	Inflammatory–lipid (prostanoid)	COX-1/COX-2, prostaglandin pathways	Potential attenuation of chronic inflammation and glial activation
Cyanidin-3-glucoside (C3G)	Inflammatory reduction	COX-2 redox enzymes (NOX4, XDH, CD38)	Modulation of inflammatory and oxidative stress in the retina
EGCG	Stress-adaptive/signaling	MAPK14, BCL2, STAT1, DNMT1	Regulation of cellular stress, apoptosis, and transcriptional adaptation
DHA/EPA	Lipid signaling and membrane stability	Polyunsaturated fatty acids, eicosanoids	Maintenance of membrane function and neuronal resistance
Coenzyme Q10	Mitochondrial-redox	Redox and metabolic enzymes, antioxidant defense	Maintenance of energy balance and protection against ROS
N-acetylcysteine (NAC)	Epigenetic/enzymatic	Histone demethylases, 2-OG dioxygenases	Potential regulation of transcriptional programs for cellular resilience

Note: The axes were defined based on PPI and functional enrichment analyses and reflect predominant, rather than exclusive, mechanisms.

It should be emphasized that the present findings are derived from computational predictions and network analyses; therefore, the identified pathways and regulatory nodes should be interpreted as hypothesis-generating observations that require further experimental validation. Key hub proteins identified in the individual networks further support the involvement of these pathways, as many of them are known regulators of inflammatory, metabolic, or signaling processes relevant to retinal neurodegeneration.

## 5. Conclusions

The present in silico study suggests that the investigated nutraceutical compounds, characterized by antioxidant and potential neuroprotective properties, may exhibit functional convergence within interconnected molecular networks implicated in the pathogenesis of glaucoma-related optic nerve degeneration. The analysis indicates possible involvement of inflammatory–lipid signaling pathways, mitochondrial redox regulation, nuclear receptor-mediated mechanisms, and epigenetically relevant processes. These findings should be interpreted as a hypothesis-generating conceptual framework that requires further experimental and clinical validation. The proposed model may serve as a basis for future investigations exploring combined adjuvant strategies in glaucoma.

## Figures and Tables

**Table 1:** Molecular formulas and SMILES codes of the included bioactive compounds.

Compound	SMILES Codes
Astaxanthin	CC1=C(C(C[C@@H](C1=O)O)(C)C)/C=C/C(=C/C=C/C(=C/C=C/C=C(/C=C/C=C(/C=C/C2=C(C(=O)[C@H](CC2(C)C)O)C)\C)\C)/C)/C
Alpha-lipoic acid (ALA)	C1CSSC1CCCCC(=O)O
Cyanidin-3-glucoside (C3G)	C1=CC(=C(C=C1C2=[O+]C3=CC(=CC(=C3C=C2O[C@H]4[C@@H]([C@H]([C@@H]([C@H](O4)CO)O)O)O)O)O)O)O
Epigallocatechin-3-gallate (EGCG)	C1[C@H]([C@H](OC2=CC(=CC(=C21)O)O)C3=CC(=C(C(=C3)O)O)O)OC(=O)C4=CC(=C(C(=C4)O)O)O
Docosahexaenoic acid (DHA)	CC/C=C\C/C=C\C/C=C/C/C=C\C/C=C\C/C=C\CCC(=O)O
Eicosapentaenoic acid (EPA)	CC/C=C/C/C=C/C/C=C/C/C=C/C/C=C/CCCC(=O)O
Coenzyme Q10	CC1=C(C(=O)C(=C(C1=O)OC)OC)C/C=C(\C)/CC/C=C(\C)/CC/C=C(\C)/CC/C=C(\C)/CC/C=C(\C)/CC/C=C(\C)/CC/C=C(\C)/CC/C=C(\C)/CC/C=C(\C)/CCC=C(C)C
N-acetyl cysteine (NAC)	CC(=O)N[C@@H](CS)C(=O)O

**Table 2:** Predicted molecular targets of selected nutraceuticals and their functional classification.

Nutraceutical	Number of Analyzed Targets	Predominant Target Classes	Key Molecular Targets (Representative)	Biological/Functional Relevance
Astaxanthin	15	Nuclear receptors, enzymes involved in steroid and lipid metabolism	AR, NR3C1, NR3C2, PGR, CYP19A1, ALOX12, CYP17A1	Nuclear–receptor regulation, steroid and lipid metabolism, inflammatory signaling
α-lipoic acid (ALA)	15	Enzymes, GPCR, nuclear receptors	PTGS1, PTGS2, PPARG, AKR1B1, CXCL8	Inflammation, eicosanoid metabolism, oxidative stress
cyanidin 3-glucoside (C3G)	15	Enzymes, oxidoreductases, GPCRs, kinases	CD38, NOX4, AKR1B1, PTGS2, XDH	Redox homeostasis, inflammation, metabolic stress
Epigallocatechin-3-gallate (EGCG)	15	Kinases, proteases, epigenetic and signaling regulators	DNMT1, DYRK1A, MAPK14, BACE1, BCL2, STAT1	Epigenetic regulation, cell survival, signal transduction
Docosahexaenoic acid (DHA)	15	Nuclear receptors, GPCR, lipid-binding proteins	PPARG, PPARA, PPARD, RXRA/B/G, FFAR1	Lipid metabolism, nuclear receptor signaling
Eicosapentaenoic acid (EPA)	15	Nuclear receptors, enzymes, lipid-binding proteins	PPARG, PPARA, ALOX5, PTGS1, FABP1	Anti-inflammatory lipid signaling
CoenzymeQ10	15	Oxidoreductases, CYP enzymes, transporters	ALOX5, CYP3A4, CYP2C9, PLA2G2A, APEX1	Mitochondrial and redox regulation
N-acetyl cysteine (NAC)	15	Epigenetic enzymes, phosphatases, GPCR	KDM2A, KDM4E, PHF8, EGLN3	Epigenetic regulation, cellular stress and adaptation

**Table 3:** Comparative summary of PPI network topology for the analyzed nutraceuticals.

Nutraceutical	Nodes	Edges	Average Node Degree	PPI Enrichment *p*-Value
Astaxanthin	14	13	1.86	4.44 × 10^−16^
α-lipoic acid (ALA)	13	5	0.769	1.35 × 10^−4^
cyanidin 3-glucoside (C3G)	15	4	0.533	3.46 × 10^−5^
Epigallocatechin-3-gallate (EGCG)	15	10	1.33	0.000369
Docosahexaenoic acid (DHA)	14	23	3.29	<1 × 10^−16^
Eicosapentaenoic acid (EPA)	15	18	2.4	<1 × 10^−16^
CoenzymeQ10	15	14	1.87	1.27 × 10^−11^
N-acetyl cysteine (NAC)	15	1	0.133	0.147

**Table 4:** Integration of in silico–predicted molecular targets with data on tissue expression, disease associations, and functional relevance from public databases and the literature.

Gene/Protein	Nutraceutical(s)	Target Class	Reported Tissue Expression (Public Databases)	Reported Disease Associations	Functional Relevance
PPARG	DHA, EPA, ALA	Nuclear receptor	High (retina) [29,30] Detected (optic nerve)	GWAS/DisGeNET (glaucoma, neurodegeneration) [29,31]	Lipid metabolism, anti-inflammatory signaling [32]
PPARA	DHA, EPA	Nuclear receptor	Moderate–High (retina) [33,34,35,36]	Metabolic & neuroinflammatory pathways [37,38]	Fatty acid oxidation, mitochondrial regulation [39,40]
ALOX5	EPA, CoQ10	Enzyme (lipoxygenase)	Detected (retina) [41,42]	Glaucoma-related inflammatory pathways [43,44,45]	Eicosanoid synthesis, inflammation [46,47,48]
PTGS2 (COX-2)	ALA, C3G	Enzyme (cyclooxygenase)	Detected (retina, glia) [49,50]	DEG in optic neuropathy models [51,52,53]	Prostaglandin-mediated inflammation [54,55]
PTGS1 (COX-1)	ALA	Enzyme (cyclooxygenase)	Detected [49]	Inflammatory signaling [56]	Basal prostanoid metabolism [55,54]
RXRA	DHA, EPA	Nuclear receptor	High (retina) [29,57]	Retinoid signaling in retinal homeostasis [58,59]	Transcriptional regulation, lipid signaling [60,61]
FABP4	DHA, EPA	Lipid-binding protein	Detected [62,63]	Metabolic stress–related pathways [64,62]	Fatty acid transport, lipid signaling [65,66]
DNMT1	EGCG	Epigenetic regulator	Detected [67,68]	Neurodegeneration-related epigenetic studies [69,70]	DNA methylation, transcriptional control [71,72]
MAPK14 (p38α)	EGCG	Kinase	Detected [73,74]	Stress-activated signaling in RGC injury [75,76]	Inflammation, stress response [77,78]
BCL2	EGCG	Apoptosis regulator	Detected [79,80]	RGC survival pathways [79,81]	Cell survival, mitochondrial integrity [82,83]
KDM5C	NAC	Histone demethylase (JmjC)	Detected [84,85]	Epigenetic regulation in neural tissue [86,87]	Chromatin remodeling, transcription [88,89]
KDM2A	NAC	Histone demethylase (JmjC)	Detected [90,91]	Epigenetic stress-response pathways [92,93]	Epigenetic adaptation [88,89]
EGLN3	NAC	2-OG–dependent dioxygenase	Detected [94,95]	Hypoxia/stress-response studies [96,97]	Oxygen sensing, epigenetic regulation [94,98]
ALOX12	Astaxanthin	Enzyme (lipoxygenase)	Detected [99,100]	Lipid peroxidation pathways [101,102]	Lipid oxidation, ferroptosis-related processes [103,104]
NR3C1 (GR)	Astaxanthin	Nuclear receptor	High (retina) [105,106]	Glial activation, stress signaling [107,108]	Glucocorticoid-mediated transcription [109,110]
NR3C2 (MR)	Astaxanthin	Nuclear receptor	Detected [111,112]	Neuroinflammatory modulation [113,114]	Stress and inflammatory regulation [115,116]
CYP19A1	Astaxanthin	Cytochrome P450	Detected [117,118]	Steroid metabolism in neural tissue [119,120]	Steroid biosynthesis [121,122]
APEX1	CoQ10	DNA repair enzyme	Detected [123,124]	Oxidative DNA damage in neurodegeneration [123,125]	Redox balance, DNA repair [126,127]
PLA2G2A	CoQ10	Phospholipase	Detected [128,129]	Inflammatory lipid signaling [130,131]	Membrane lipid remodeling [132,133]

## Data Availability

The original contributions presented in this study are included in the article/Supplementary Materials. Further inquiries can be directed to the corresponding author.

## Supplementary Materials

The following supporting information can be downloaded at: https://www.mdpi.com/article/10.3390/antiox15040445/s1, Table S1: Detailed functional enrichment results (pathways, gene count, and FDR) for each analyzed nutraceutical.

## References

[1] Smith A.M., Czyz C.N. (2025). Neuroanatomy, Cranial Nerve 2 (Optic). StatPearls [Internet].

[2] Glisson C.C. (2014). Visual loss due to optic chiasm and retrochiasmal visual pathway lesions. Continuum.

[3] Sanz-Morello B., Ahmadi H., Vohra R., Saruhanian S., Freude K.K., Hamann S., Kolko M. (2021). Oxidative Stress in Optic Neuropathies. Antioxidants.

[4] Catalani E., Brunetti K., Del Quondam S., Cervia D. (2023). Targeting Mitochondrial Dysfunction and Oxidative Stress to Prevent Neurodegeneration of Retinal Ganglion Cells. Antioxidants.

[5] Buonfiglio F., Böhm E.W., Pfeiffer N., Gericke A. (2023). Oxidative Stress: A Suitable Therapeutic Target for Optic Nerve Diseases?. Antioxidants.

[6] Yang Y., Lin Y., Han Z., Wang B., Zheng W., Wei L. (2024). Ferroptosis: A novel mechanism of cell death in ophthalmic conditions. Front. Immunol..

[7] Ren X., Léveillard T. (2022). Modulating antioxidant systems as a therapeutic approach to retinal degeneration. Redox Biol..

[8] Usui S., Komeima K., Lee S.Y., Jo Y.-J., Ueno S., Rogers B.S., Wu Z., Shen J., Lu L., Oveson B.C. (2009). Increased expression of catalase and superoxide dismutase 2 reduces cone cell death in retinitis pigmentosa. Mol. Ther..

[9] Tham Y.C., Li X., Wong T.Y., Quigley H.A., Aung T., Cheng C.Y. (2014). Global prevalence of glaucoma and projections of glaucoma burden through 2040: A systematic review and meta-analysis. Ophthalmology.

[10] Wang L.H., Huang C.H., Lin I.C. (2024). Advances in Neuroprotection in Glaucoma. Pharmaceuticals.

[11] Kuo C.Y., Liu C.J.L. (2022). Neuroprotection in Glaucoma: Basic Aspects and Clinical Relevance. J. Pers. Med..

[12] D’Angelo A., Vitiello L., Lixi F., Abbinante G., Coppola A., Gagliardi V., Pellegrino A., Giannaccare G. (2024). Optic Nerve Neuroprotection in Glaucoma: A Narrative Review. J. Clin. Med..

[13] Bou Ghanem G.O., Wareham L.K., Calkins D.J. (2024). Addressing neurodegeneration in glaucoma: Mechanisms, challenges, and treatments. Prog. Retin. Eye Res..

[14] Garcia-Medina J.J., Rubio-Velazquez E., Lopez-Bernal M.D., Cobo-Martinez A., Zanon-Moreno V., Pinazo-Duran M.D., Del-Rio-Vellosillo M. (2020). Glaucoma and Antioxidants: Review and Update. Antioxidants.

[15] Wang M.X., Zhao J., Zhang H., Li K., Niu L.-Z., Wang Y.-P., Zheng Y.-J. (2020). Potential Protective and Therapeutic Roles of the Nrf2 Pathway in Ocular Diseases: An Update. Oxid. Med. Cell Longev..

[16] Anton A., Garcia V., Muñoz M., Gonzales K., Ayala E., Sanchez E.d.M., Morilla-Grasa A. (2022). Effect of Oral Citicoline and DHA on Visual Field in Glaucoma. Life.

[17] Falardeau J., Fryman A., Wanchu R., Marracci G.H., Mass M., Wooliscroft L., Bourdette D.N., Murchison C.F., Hills W.L., Yadav V. (2019). Oral lipoic acid for acute optic neuritis: A randomized trial. Mult. Scler. J. Exp. Transl. Clin..

[18] Inman D.M., Lambert W.S., Calkins D.J., Horner P.J. (2013). α-Lipoic acid antioxidant treatment limits glaucoma-related retinal ganglion cell death. PLoS ONE.

[19] Ohguro H., Ohguro I., Katai M., Tanaka S. (2012). Black Currant Anthocyanins on Visual Field in Glaucoma: 2-Year RCT. Ophthalmologica.

[20] Martucci A., Nucci C. (2019). Evidence on neuroprotective properties of CoQ10 in glaucoma. Neural Regen. Res..

[21] Ahmad S.S. (2020). Coenzyme Q and its role in glaucoma. Saudi J. Ophthalmol..

[22] Sano H., Namekata K., Kimura A., Shitara H., Guo X., Harada C., Mitamura Y., Harada T. (2019). NAC effects in glaucoma models. Cell Death Dis..

[23] Gawin-Mikołajewicz A., Nartowski K.P., Dyba A.J., Gołkowska A.M., Malec K., Karolewicz B. (2021). Ophthalmic Nanoemulsions: Composition to Quality Control. Mol. Pharm..

[24] Batur E., Özdemir S., Durgun M.E., Özsoy Y. (2024). Vesicular Drug Delivery Systems in Ocular Therapy. Pharmaceuticals.

[25] Terao K., Nakata D., Fukumi H., Schmid G., Arima H., Hirayama F., Uekama K. (2006). Oral bioavailability of CoQ10 complexed with γ-cyclodextrin. Nutr. Res..

[26] Pravst I., Rodríguez Aguilera J.C., Cortes Rodríguez A.B., Jazbar J., Locatelli I., Hristov H., Žmitek K. (2020). Bioavailability of CoQ10 formulations in elderly. Nutrients.

[27] Gfeller D., Grosdidier A., Wirth M., Daina A., Michielin O., Zoete V. (2014). SwissTargetPrediction: A web server for target prediction of bioactive small molecules. Nucleic Acids Res..

[28] Szklarczyk D., Kirsch R., Koutrouli M., Nastou K., Mehryary F., Hachilif R., Gable A.L., Fang T., Doncheva N.T., Pyysalo S. (2023). The STRING database in 2023: Protein-protein association networks and functional enrichment analyses for any sequenced genome of interest. Nucleic Acids Res..

[29] Choudhary M., Malek G. (2016). Rethinking Nuclear Receptors as Potential Therapeutic Targets for Retinal Diseases. J. Biomol. Screen..

[30] Liu K., Zou C., Qin B. (2017). The association between nuclear receptors and ocular diseases. Oncotarget.

[31] Escandon P., Vasini B., Whelchel A.E., Nicholas S.E., Matlock H.G., Ma J.X., Karamichos D. (2021). The role of peroxisome proliferator-activated receptors in healthy and diseased eyes. Exp. Eye Res..

[32] Zhu C.-Y., Yu P.-H., Sun Q., Hong D.-F., Yang C., Naranmandura H. (2025). Nuclear receptors in metabolism and diseases: Mechanistic and therapeutic insights. Pharmacol. Res..

[33] Yao F., Zhang X., Yao X., Ren X., Xia X., Jiang J., Ding L. (2021). Peroxisome Proliferator-Activated Receptor α Activation Protects Retinal Ganglion Cells in Ischemia-Reperfusion Retinas. Front. Med..

[34] Pearsall E.A., Cheng R., Matsuzaki S., Zhou K., Ding L., Ahn B., Kinter M., Humphries K.M., Quiambao A.B., Farjo R.A. (2019). Neuroprotective effects of PPARα in retinopathy of type 1 diabetes. PLoS ONE.

[35] Moran E., Ding L., Wang Z., Cheng R., Chen Q., Moore R., Takahashi Y., Ma J.-X. (2014). Protective and Antioxidant Effects of PPAR in the Ischemic Retina. Investig. Ophthalmol. Vis. Sci..

[36] Roy S., Kern T.S., Song B., Stuebe C. (2017). Mechanistic Insights into Pathological Changes in the Diabetic Retina: Implications for Targeting Diabetic Retinopathy. Am. J. Pathol..

[37] Bensinger S.J., Tontonoz P. (2008). Integration of metabolism and inflammation by lipid-activated nuclear receptors. Nature.

[38] King J.L., Smithers L., Vrielink A., Lesterhuis W.J., Piggott M.J. (2025). A New Era for PPARγ: Covalent Ligands and Therapeutic Applications. J. Med. Chem..

[39] Kersten S. (2014). Integrated physiology and systems biology of PPARα. Mol. Metab..

[40] Pawlak M., Lefebvre P., Staels B. (2015). Molecular mechanism of PPARα action and its impact on lipid metabolism, inflammation and fibrosis in non-alcoholic fatty liver disease. J. Hepatol..

[41] Trotta M.C., Gesualdo C., Petrillo F., Lepre C.C., Della Corte A., Cavasso G., Maggiore G., Hermenean A., Simonelli F., D’amico M. (2022). Resolution of Inflammation in Retinal Disorders: Briefly the State. Int. J. Mol. Sci..

[42] Jayawardana S.A.S., Samarasekera J.K.R.R., Hettiarachchi G.H.C.M., Gooneratne M.J. (2025). The pathogenetic roles of arachidonate 5-lipoxygenase, xanthine oxidase and hyaluronidase in inflammatory diseases: A review. J. Biomed. Res..

[43] Tezel G. (2013). Immune regulation toward immunomodulation for neuroprotection in glaucoma. Curr. OpinPharmacol.

[44] Howell G.R., Macalinao D.G., Sousa G.L., Walden M., Soto I., Kneeland S.C., Barbay J.M., King B.L., Marchant J.K., Hibbs M. (2011). Molecular clustering identifies complement and endothelin induction as early events in a mouse model of glaucoma. J. Clin. Investig..

[45] Okruszko M.A., Szabłowski M., Zarzecki M., Michnowska-Kobylińska M., Lisowski Ł., Łapińska M., Stachurska Z., Szpakowicz A., Kamiński K.A., Konopińska J. (2024). Inflammation and Neurodegeneration in Glaucoma: Isolated Eye Disease or a Part of a Systemic Disorder?—Serum Proteomic Analysis. J. Inflamm. Res..

[46] Peters-Golden M., Henderson W.R. (2007). Leukotrienes. N. Engl. J. Med..

[47] Rådmark O., Samuelsson B. (2009). 5-Lipoxygenase: Mechanisms of regulation. J. Lipid Res..

[48] Haeggström J.Z., Funk C.D. (2011). Lipoxygenase and Leukotriene Pathways: Biochemistry, Biology, and Roles in Disease. Chem. Rev..

[49] Neufeld A.H., Hernandez M.R., Gonzalez M., Geller A. (1997). Cyclooxygenase-1 and cyclooxygenase-2 in the human optic nerve head. Exp. Eye Res..

[50] Wang X., Tay S.S., Ng Y.K. (2000). An immunohistochemical study of neuronal and glial cell reactions in retinae of rats with experimental glaucoma. Exp. Brain Res..

[51] Vernazza S., Tirendi S., Bassi A.M., Traverso C.E., Saccà S.C. (2020). Neuroinflammation in Primary Open-Angle Glaucoma. J. Clin. Med..

[52] Vohra R., Tsai J.C., Kolko M. (2013). The Role of Inflammation in the Pathogenesis of Glaucoma. Surv. Ophthalmol..

[53] Yang X., Hondur G., Tezel G. (2016). Antioxidant Treatment Limits Neuroinflammation in Experimental Glaucoma. Invest. Ophthalmol. Vis. Sci..

[54] Smith W.L., DeWitt D.L., Garavito R.M. (2000). Cyclooxygenases: Structural, Cellular, and Molecular Biology. Annu. Rev. Biochem..

[55] Funk C.D. (2001). Prostaglandins and leukotrienes: Advances in eicosanoid biology. Science.

[56] Smith W.L., Garavito R.M., DeWitt D.L. (1996). Prostaglandin Endoperoxide H Synthases (Cyclooxygenases)-1 and −2. J. Biol. Chem..

[57] Zhou J.Y., Ong E.S., Oro A.E., Kakizuka A., Evans R.M. (1992). Characterization of three RXR genes that mediate the action of 9-cis retinoic acid. Genes. Dev..

[58] Duester G. (2008). Retinoic acid synthesis and signaling during early organogenesis. Cell.

[59] Ghyselinck N.B., Duester G. (2019). Retinoic acid signaling pathways. Development.

[60] Evans R.M., Mangelsdorf D.J. (2014). Nuclear Receptors, RXR, and the Big Bang. Cell.

[61] Beato M. (1991). Transcriptional control by nuclear receptors. FASEB J..

[62] Furuhashi M., Hotamisligil G.S. (2008). Fatty acid-binding proteins: Role in metabolic diseases and potential as drug targets. Nat. Rev. Drug Discov..

[63] Matsumata M., Inada H., Osumi N. (2016). Fatty acid binding proteins and the nervous system: Their impact on mental conditions. Neurosci. Res..

[64] Hotamisligil G.S. (2017). Foundations of Immunometabolism and Implications for Metabolic Health and Disease. Immunity.

[65] Storch J., McDermott L. (2009). Structural and The functional analysis of fatty acid-binding proteins. J. Lipid Res..

[66] Glatz J.F., van der Vusse G.J. (1996). Cellular fatty acid-binding proteins: Their function and physiological significance. Prog. Lipid Res..

[67] Feng J., Chang H., Li E., Fan G. (2005). Dynamic expression of de novo DNA methyltransferases Dnmt3a and Dnmt3b in the central nervous system. J. Neurosci. Res..

[68] Lister R., Mukamel E.A., Nery J.R., Urich M., Puddifoot C.A., Johnson N.D., Lucero J., Huang Y., Dwork A.J., Schultz M.D. (2013). Global epigenomic reconfiguration during mammalian brain development. Science.

[69] Qureshi I.A., Mehler M.F. (2018). Epigenetic mechanisms underlying nervous system diseases. Handb. Clin. Neurol..

[70] Jakovcevski M., Akbarian S. (2012). Epigenetic mechanisms in neurological disease. Nat. Med..

[71] Bird A. (2002). DNA methylation patterns and epigenetic memory. Genes. Dev..

[72] Bestor T.H. (2000). The DNA methyltransferases of mammals. Hum. Mol. Genet..

[73] Gräb J., Rybniker J. (2019). The Expanding Role of p38 Mitogen-Activated Protein Kinase in Programmed Host Cell Death. Microbiol. Insights.

[74] Katome T., Namekata K., Guo X., Semba K., Kittaka D., Kawamura K., Kimura A., Harada C., Ichijo H., Mitamura Y. (2013). Inhibition of ASK1-p38 pathway prevents neural cell death following optic nerve injury. Cell Death Differ..

[75] Tezel G., Yang X. (2005). Comparative gene array analysis of TNF-alpha-induced MAPK and NF-kappaB signaling pathways between retinal ganglion cells and glial cells. Exp. Eye Res..

[76] Campos C.B., Bédard P.A., Linden R. (2006). Requirement of p38 stress-activated MAP kinase for cell death in the developing retina depends on the stage of cell differentiation. Neurochem. Int..

[77] Kyriakis J.M., Avruch J. (2012). Mammalian MAPK signal transduction pathways activated by stress and inflammation: A 10-year update. Physiol. Rev..

[78] Ono K., Han J. (2000). The p38 signal transduction pathway: Activation and function. Cell Signal..

[79] Nickells R.W., Zack D.J. (1996). Apoptosis in ocular disease: A molecular overview. Ophthalmic Genet..

[80] Levin L.A., Gordon L.K. (2002). Retinal ganglion cell disorders: Types and treatments. Prog. Retin. Eye Res..

[81] Bonfanti L., Strettoi E., Chierzi S., Cenni M.C., Liu X.H., Martinou J.-C., Maffei L., Rabacchi S.A. (1996). Protection of retinal ganglion cells from natural and axotomy-induced cell death in neonatal transgenic mice overexpressing bcl-2. J. Neurosci..

[82] Youle R.J., Strasser A. (2008). The BCL-2 protein family: Opposing activities that mediate cell death. Nat. Rev. Mol. Cell Biol..

[83] Cory S., Adams J.M. (2002). The Bcl2 family: Regulators of the cellular life-or-death switch. Nat. Rev. Cancer.

[84] Iwase S., Lan F., Bayliss P., de la Torre-Ubieta L., Huarte M., Qi H.H., Whetstine J.R., Bonni A., Roberts T.M., Shi Y. (2007). The X-linked mental retardation gene SMCX/JARID1C defines a family of histone H3 lysine 4 demethylases. Cell.

[85] Chen Q., Chen X., Wang Q., Zhang F., Lou Z., Zhang Q., Zhou D.-X. (2013). Structural basis of a histone H3 lysine 4 demethylase required for stem elongation in rice. PLoS Genet..

[86] Vallianatos C.N., Iwase S. (2015). Disrupted intricacy of histone H3K4 methylation in neurodevelopmental disorders. Epigenomics.

[87] Scandaglia M., Lopez-Atalaya J.P., Medrano-Fernandez A., Lopez-Cascales M.T., del Blanco B., Lipinski M., Benito E., Olivares R., Iwase S., Shi Y. (2017). Loss of Kdm5c Causes Spurious Transcription and Prevents the Fine-Tuning of Activity-Regulated Enhancers in Neurons. Cell Rep..

[88] Klose R.J., Zhang Y. (2007). Regulation of histone methylation by demethylimination and demethylation. Nat. Rev. Mol. Cell Biol..

[89] Pedersen M.T., Helin K. (2010). Histone demethylases in development and disease. Trends Cell Biol..

[90] Ren Z., Tang H., Zhang W., Guo M., Cui J., Wang H., Xie B., Yu J., Chen Y., Zhang M. (2024). The Role of KDM2A and H3K36me2 Demethylation in Modulating MAPK Signaling During Neurodevelopment. Neurosci. Bull..

[91] Kawakami E., Tokunaga A., Ozawa M., Sakamoto R., Yoshida N. (2015). The histone demethylase Fbxl11/Kdm2a plays an essential role in embryonic development by repressing cell-cycle regulators. Mech. Dev..

[92] Tsukada Y., Fang J., Erdjument-Bromage H., Warren M.E., Borchers C.H., Tempst P., Zhang Y. (2006). Histone demethylation by a family of JmjC domain-containing proteins. Nature.

[93] Blackledge N.P., Klose R.J. (2021). The molecular principles of gene regulation by Polycomb repressive complexes. Nat. Rev. Mol. Cell Biol..

[94] Kaelin W.G., Ratcliffe P.J. (2008). Oxygen Sensing by Metazoans: The Central Role of the HIF Hydroxylase Pathway. Mol. Cell.

[95] Appelhoff R.J., Tian Y.M., Raval R.R., Turley H., Harris A.L., Pugh C.W., Ratcliffe P.J., Gleadle J.M. (2004). Differential function of the prolyl hydroxylases PHD1, PHD2, and PHD3 in the regulation of hypoxia-inducible factor. J. Biol. Chem..

[96] Schlisio S. (2009). Neuronal apoptosis by prolyl hydroxylation: Implication in nervous system tumours and the Warburg conundrum. J. Cell Mol. Med..

[97] Myllyharju J. (2013). Prolyl 4-hydroxylases, master regulators of the hypoxia response. Acta Physiol..

[98] Batie M., Rocha S. (2020). Gene transcription and chromatin regulation in hypoxia. Biochem. Soc. Trans..

[99] Brash A.R. (1999). Lipoxygenases: Occurrence, Functions, Catalysis, and Acquisition of Substrate. J. Biol. Chem..

[100] Al-Shabrawey M., Mussell R., Kahook K., Tawfik A., Eladl M., Sarthy V., Nussbaum J., El-Marakby A., Park S.Y., Gurel Z. (2011). Increased expression and activity of 12-lipoxygenase in oxygen-induced ischemic retinopathy and proliferative diabetic retinopathy: Implications in retinal neovascularization. Diabetes.

[101] Cakir-Aktas C., Bodur E., Yemisci M., van Leyen K., Karatas H. (2023). 12/15-lipoxygenase inhibition attenuates neuroinflammation by suppressing inflammasomes. Front. Cell Neurosci..

[102] Shintoku R., Takigawa Y., Yamada K., Kubota C., Yoshimoto Y., Takeuchi T., Koshiishi I., Torii S. (2017). Lipoxygenase-mediated generation of lipid peroxides enhances ferroptosis induced by erastin and RSL3. Cancer Sci..

[103] Yang W.S., Stockwell B.R. (2016). Ferroptosis: Death by Lipid Peroxidation. Trends Cell Biol..

[104] Kagan V.E., Mao G., Qu F., Angeli J.P., Doll S., Croix C.S., Dar H.H., Liu B., Tyurin V.A., Ritov V.B. (2017). Oxidized arachidonic and adrenic PEs navigate cells to ferroptosis. Nat. Chem. Biol..

[105] Gallina D., Zelinka C., Fischer A.J. (2014). Glucocorticoid receptors in the retina, Müller glia and the formation of Müller glia-derived progenitors. Development.

[106] Sulaiman R.S., Kadmiel M., Cidlowski J.A. (2018). Glucocorticoid receptor signaling in the eye. Steroids.

[107] Rashid K., Akhtar-Schaefer I., Langmann T. (2019). Microglia in Retinal Degeneration. Front. Immunol..

[108] Sorrells S.F., Sapolsky R.M. (2007). An inflammatory review of glucocorticoid actions in the CNS. Brain Behav. Immun..

[109] Beato M., Herrlich P., Schütz G. (1995). Steroid hormone receptors: Many actors in search of a plot. Cell.

[110] Oakley R.H., Cidlowski J.A. (2013). The biology of the glucocorticoid receptor: New signaling mechanisms in health and disease. J. Allergy Clin. Immunol..

[111] Reul J.M., de Kloet E.R. (1985). Two receptor systems for corticosterone in rat brain: Microdistribution and differential occupation. Endocrinology.

[112] Behar-Cohen F., Zhao M. (2022). Mineralocorticoid pathway in retinal health and diseases. Br. J. Pharmacol..

[113] de Kloet E.R., Joëls M., Holsboer F. (2005). Stress and the brain: From adaptation to disease. Nat. Rev. Neurosci..

[114] Dougherty E.J., Elinoff J.M., Ferreyra G.A., Hou A., Cai R., Sun J., Blaine K.P., Wang S., Danner R.L. (2016). Mineralocorticoid Receptor (MR) trans-Activation of Inflammatory AP-1 Signaling: DEPENDENCE ON DNA SEQUENCE, MR CONFORMATION, AND AP-1 FAMILY MEMBER EXPRESSION. J. Biol. Chem..

[115] de Kloet E.R., Joëls M. (2017). Brain mineralocorticoid receptor function in control of salt balance and stress-adaptation. Physiol. Behav..

[116] Gomez-Sanchez E., Gomez-Sanchez C.E. (2014). The multifaceted mineralocorticoid receptor. Compr. Physiol..

[117] Simpson E.R., Misso M., Hewitt K.N., Hill R.A., Boon W.C., Jones M.E., Kovacic A., Zhou J., Clyne C.D. (2005). Estrogen—The Good, the Bad, and the Unexpected. Endocr. Rev..

[118] Azcoitia I., Mendez P., Garcia-Segura L.M. (2021). Aromatase in the Human Brain. Androg. Clin. Res. Ther..

[119] Garcia-Segura L.M., Azcoitia I., DonCarlos L.L. (2001). Neuroprotection by estradiol. Prog. Neurobiol..

[120] McCarthy M.M. (2008). Estradiol and the developing brain. Physiol. Rev..

[121] Simpson E.R., Clyne C., Rubin G., Boon W.C., Robertson K., Britt K., Speed C., Jones M. (2002). Aromatase—A brief overview. Annu. Rev. Physiol..

[122] Conley A., Hinshelwood M. (2001). Mammalian aromatases. Reproduction.

[123] Madabhushi R., Pan L., Tsai L.H. (2014). DNA damage and its links to neurodegeneration. Neuron.

[124] Vasko M.R., Guo C., Kelley M.R. (2005). The multifunctional DNA repair/redox enzyme Ape1/Ref-1 promotes survival of neurons after oxidative stress. DNA Repair..

[125] Fishel M.L., Kelley M.R. (2007). The DNA base excision repair protein Ape1/Ref-1 as a therapeutic and chemopreventive target. Mol. Asp. Med..

[126] Tell G., Quadrifoglio F., Tiribelli C., Kelley M.R. (2009). The many functions of APE1/Ref-1: Not only a DNA repair enzyme. Antioxid. Redox Signal..

[127] Xanthoudakis S., Curran T. (1992). Identification and characterization of Ref-1, a nuclear protein that facilitates AP-1 DNA-binding activity. EMBO J..

[128] Moses G.S., Jensen M.D., Lue L.F., Walker D.G., Sun A.Y., Simonyi A., Sun G.Y. (2006). Secretory PLA2-IIA: A new inflammatory factor for Alzheimer’s disease. J. Neuroinflammation..

[129] Sun G.Y., Shelat P.B., Jensen M.B., He Y., Sun A.Y., Simonyi A. (2010). Phospholipases A2 and inflammatory responses in the central nervous system. Neuromolecular Med..

[130] Dennis E.A., Norris P.C. (2015). Eicosanoid storm in infection and inflammation. Nat. Rev. Immunol..

[131] Murakami M., Taketomi Y., Girard C., Yamamoto K., Lambeau G. (2010). Emerging roles of secreted phospholipase A2 enzymes: Lessons from transgenic and knockout mice. Biochimie.

[132] Six D.A., Dennis E.A. (2000). The expanding superfamily of phospholipase A(2) enzymes: Classification and characterization. Biochim. Biophys. Acta.

[133] Burke J.E., Dennis E.A. (2009). Phospholipase A2 structure/function, mechanism, and signaling. J. Lipid Res..

[134] Qi T., Liu H., Frühn L., Löw K., Cursiefen C., Prokosch V. (2025). Understanding Glaucoma: Why it Remains a Leading Cause of Blindness Worldwide. Klin. Monbl Augenheilkd..

[135] Sun Y., Chen A., Zou M., Zhang Y., Jin L., Li Y., Zheng D., Jin G., Congdon N. (2022). Time trends, associations and prevalence of blindness and vision loss due to glaucoma: An analysis of observational data from the Global Burden of Disease Study 2017. BMJ Open.

[136] Liu Z., Ang G.S. (2025). Nutraceuticals and neuroprotection for glaucoma-introducing the NP-10 System. Ther. Adv. Ophthalmol..

[137] Adornetto A., Rombolà L., Morrone L.A., Nucci C., Corasaniti M.T., Bagetta G., Russo R. (2020). Natural Products: Evidence for Neuroprotection to Be Exploited in Glaucoma. Nutrients.

[138] Loskutova E., O’Brien C., Loskutov I., Loughman J. (2018). Nutritional supplementation in the treatment of glaucoma: A systematic review. Surv. Ophthalmol..

[139] Wei Y., Lin Y., Li Y., Liu J., Yang Y., Chen H., Han Z., Wang K., Qian T., Ju Y. (2025). Redefining cell death: Ferroptosis as a game-changer in ophthalmology. Front. Immunol..

[140] Wood J.P.M., Chidlow G., Wall G.M., Casson R.J. (2024). N-acetylcysteine amide and di- N-acetylcysteine amide protect retinal cells in culture via an antioxidant action. Exp. Eye Res..

[141] Zhu Y., Moksha L., Salowe R., Vrathasha V., Pham K., Aibo M.-A.I., Lee R., Halimitabrizi M., Di Rosa I., O’Brien J.M. (2025). Integrating neuroprotection, antioxidative effects, and precision medicine in glaucoma management with bioactive compounds. Biomed. Pharmacother..

[142] Wu S., Jiang H., Chen Z., Lu W., Chen Q. (2022). Network Pharmacology-Based Study on the Active Ingredients and Mechanism of Pan Ji Sheng Traditional Chinese Medicine Formula in the Treatment of Inflammation. Evid.-Based Complement. Altern. Med..

[143] Geiduschek E.K., McDowell C.M. (2023). The Fibro-Inflammatory Response in the Glaucomatous Optic Nerve Head. Int. J. Mol. Sci..

[144] Aggarwal B.B., Van Kuiken M.E., Iyer L.H., Harikumar K.B., Sung B. (2009). Molecular targets of nutraceuticals derived from dietary spices: Potential role in suppression of inflammation and tumorigenesis. Exp. Biol. Med..

[145] Yang T.-H., Kang E.Y.-C., Lin P.-H., Yu B.B.-C., Wang J.H.-H., Chen V., Wang N.-K. (2024). Mitochondria in Retinal Ganglion Cells: Unraveling the Metabolic Nexus and Oxidative Stress. Int. J. Mol. Sci..

[146] Muench N.A., Patel S., Maes M.E., Donahue R.J., Ikeda A., Nickells R.W. (2021). The Influence of Mitochondrial Dynamics and Function on Retinal Ganglion Cell Susceptibility in Optic Nerve Disease. Cells.

[147] Lewis L.S.C., Skiba N.P., Hao Y., Bomze H.M., Arshavsky V.Y., Cartoni R., Gospe S.M. (2024). Compartmental Differences in the Retinal Ganglion Cell Mitochondrial Proteome. bioRxiv.

[148] Sykiotis G.P., Papavassiliou A.G. (2002). Molecular mechanisms of transcriptional regulation by nuclear receptors. Perspectives for therapeutic implications. Hormones.

[149] Krause B.J., Paz A.A., Garrud T.A.C., Peñaloza E., Vega-Tapia F., Ford S.G., Niu Y., Giussani D.A. (2024). Epigenetic regulation by hypoxia, N-acetylcysteine and hydrogen sulphide of the fetal vasculature in growth restricted offspring: A study in humans and chicken embryos. J. Physiol..

[150] Peña C.J. (2026). Epigenetic regulation of brain development, plasticity, and response to early-life stress. Neuropsychopharmacology.

[151] Pastore A., Badolati N., Manfrevola F., Sagliocchi S., Laurenzi V., Musto G., Porreca V., Murolo M., Chioccarelli T., Ciampaglia R. (2024). N-acetyl-L-cysteine reduces testis ROS in obese fathers but fails in protecting offspring from acquisition of epigenetic traits at cyp19a1 and IGF11/H19 ICR loci. Front. Cell Dev. Biol..

