# Supplementary material for: Network Analysis of Convergent and Specific Molecular Pathways of Nutraceuticals with Antioxidant and Neuroprotective Potential in Glaucoma
Source: Antioxidants (Basel). 2026 Apr 2;15(4):445. doi: 10.3390/antiox15040445 (PMC13113343; doi:10.3390/antiox15040445)
Supplement: Supplementary file 1 [file antioxidants-15-00445-s001.zip › antioxidants-4180698-supplementary.pdf]

Supplementary Table S1

Compound	Pathway	Gene count	FDR
Astaxanthin	Intracellular steroid hormone receptor signaling pathway	4	7.8×10^{-4}
Astaxanthin	Glucocorticoid metabolic process	3	1.2×10^{-3}
Astaxanthin	Steroid metabolic process	5	1.9×10^{-3}
Astaxanthin	Lipid metabolic process	8	1.4×10^{-3}
Astaxanthin	Mammary gland development	4	3.8×10^{-3}
ALA	Cyclooxygenase pathway	3	2.2×10^{-4}
ALA	Prostaglandin metabolic process	4	2.6×10^{-4}
ALA	Olefinic compound metabolic process	4	2.0×10^{-3}
ALA	Monocarboxylic acid biosynthetic process	4	2.3×10^{-3}
ALA	Fatty acid metabolic process	5	3.1×10^{-3}
CG3	One-carbon metabolic process	4	4.1×10^{-4}
CG3	Regulation of smooth muscle contraction	4	1.1×10^{-3}
CG3	Regulation of vasoconstriction	4	1.3×10^{-3}
CG3	Modulation of chemical synaptic transmission	6	1.7×10^{-3}
CG3	Small molecule metabolic process	9	1.7×10^{-3}
EGCG	Amyloid fibril formation	3	1.2×10^{-3}
EGCG	Response to lead ion	3	1.2×10^{-3}
EGCG	Positive regulation of smooth muscle cell proliferation	4	2.2×10^{-3}
EGCG	Regulation of vascular associated smooth muscle cell proliferation	3	2.4×10^{-3}

EGCG	Positive regulation of striated muscle cell differentiation	3	2.7×10^{-3}
DHA	Retinoic acid receptor signaling pathway	6	7.73×10^{-11}
DHA	Intracellular receptor signaling pathway	8	2.90×10^{-10}
DHA	Hormone-mediated signaling pathway	7	1.01×10^{-8}
DHA	Response to vitamin A	4	1.99×10^{-6}
DHA	Negative regulation of miRNA transcription	4	2.19×10^{-6}
EPA	Regulation of fatty acid oxidation	6	4.98×10^{-9}
EPA	Regulation of fatty acid metabolic process	7	4.98×10^{-9}
EPA	Fatty acid transport	6	7.59×10^{-8}
EPA	Positive regulation of fatty acid oxidation	4	2.73×10^{-6}
EPA	Long-chain fatty acid transport	5	2.03×10^{-6}
CoQ 10	Monoterpenoid metabolic process	4	3.56×10^{-7}
CoQ10	Olefinic compound metabolic process	7	1.29×10^{-7}
CoQ10	Xenobiotic catabolic process	4	2.66×10^{-5}
CoQ10	Long-chain fatty acid biosynthetic process	4	3.02×10^{-5}
CoQ10	Estrogen metabolic process	4	3.87×10^{-5}
NAC	Histone lysine demethylation	5	4.45×10^{-7}
NAC	Histone H3-K36 demethylation	3	2.7×10^{-4}
NAC	Histone H3-K9 demethylation	3	5.4×10^{-4}
NAC	Histone H4-K20 demethylation	2	9.4×10^{-3}
NAC	Histone H3-K27 demethylation	2	1.48×10^{-2}
